# Risk factors for complications and readmission after operative fixation of pediatric femur fractures

**DOI:** 10.1007/s11832-015-0672-x

**Published:** 2015-08-05

**Authors:** Amit Momaya, Dustin Baker, Shawn Gilbert, Brent Ponce

**Affiliations:** University of Alabama at Birmingham, 1313 13th Street South, Birmingham, AL 35205 USA

**Keywords:** Pediatric femur fracture, Risk factors, Complications, Readmission

## Abstract

**Purpose:**

Operative fixation of pediatric femur fractures with intramedullary implants has grown in popularity in recent decades. However, risk factors for short-term adverse events and readmission have not been well studied.

**Methods:**

Pediatric patients who underwent intramedullary nailing of a femur fracture between 2012 and 2013 were identified from the American College of Surgeons National Surgical Quality Improvement Program database. Risk factors for any adverse event (AAE) and readmission after intramedullary nailing were evaluated using univariate and multivariate analysis.

**Results:**

A total of 522 pediatric patients who underwent intramedullary nailing of the femur during the study period were identified. The mean age of this patient cohort was 10.2 ± 3.8 years. Review of the cases revealed that 18 (3.4 %) patients had AAE and that 20 (3.8 %) patients were readmitted, of whom 13 (2.5 %) underwent a reoperation. Independent risk factors for AAE were a cardiac comorbidity [odds ratio (OR) 12.7, 95 % confidence interval (CI) 1.5, 103.7], open fracture (OR 10.2, 95 % CI 1.4, 74.4), and prolonged operative time (OR 17.5, 95 % CI 6.1, 50.5). Independent risk factors for readmission were a central nervous system disorder (OR 4.5, 95 % CI 1.3, 16.2) and a seizure disorder (OR 4.9, 95 % CI 1.0, 23.5).

**Conclusions:**

The results of the multivariate analysis suggest that cardiac comorbidities, open fractures, and prolonged operative time increase the risk for AAE and that central nervous system disorders and seizure disorders may increase the risk for readmission. Surgeons should be aware of these risk factors and counsel the families of pediatric patients who undergo intramedullary nailing of femur fractures.

## Introduction

Operative treatment of pediatric femur fractures, especially with intramedullary implants, has grown increasingly popular in recent years [1–5]. Successful outcomes of this operative treatment have been well documented [6–11], with the benefits including earlier mobilization, shorter hospital stays, and earlier return to school [12, 13].

Although generally considered to be a safe procedure, complications have been reported after the fixation of pediatric femur fractures with intramedullary implants and include pain at nail insertion site, nail prominence, superficial and deep infections, mal-angulation, hematoma, neurologic damage, delayed union or nonunion, and loss of reduction [4, 14, 15]. Previously reported risk factors for complications and poor outcomes include older age, increased weight [3, 4], improper sizing of the nail [16], and type of nail (rigid antegrade vs. elastic retrograde) [5].

The American College of Surgeons National Surgical Quality Improvement Program (ACS NSQIP) database collects data prospectively on patient outcomes in the 30-day period after the procedure. Patient demographics, comorbidities, and complications are recorded by the assigned NSQIP staff in 8-day cycles. The NSQIP database has the important advantage of having a larger sample size of patients than could ever be obtained by a single institution. Given the overall safety and low incidence of adverse events and readmission after operative fixation of pediatric femur fractures, this database provides a good platform for studying potential risk factors associated with this procedure.

The purpose of our study was to identify risk factors for short-term adverse events and readmission after the operative fixation of pediatric femur fractures with intramedullary implants. Our hypothesis was that medical comorbidities, obesity, and open fractures would serve as risk factors for complications and readmission.

## Materials and methods

Approval from the institutional review board at our institution was not required for this study. The ACS NSQIP database is de-identified and publicly available.

A search query was performed in the ACS NSQIP database for pediatric patients between the ages of 4 and 18 years who underwent operative treatment of femur fractures with intramedullary implants from 2012 to 2013. Pediatric patients under the age of 4 years were excluded because operative fixation of a femur fracture in this population is often due to an unusual indication, such as a pathologic fracture or osteogenesis imperfecta. Patients above 4 years of age with a primary diagnosis of pathologic fracture or osteogenesis imperfecta were also excluded. Current Procedural Terminology code 27506 (open treatment of femoral shaft fracture, with or without external fixation, with insertion of intramedullary implant, with or without cerclage and/or locking screws) was used.

Data on any adverse event (AAE) were collected. AAE included superficial surgical site infection (SSI), deep SSI, organ space SSI, wound dehiscence, postoperative pneumonia, unplanned reintubation, pulmonary embolism, deep venous thromboembolism or thrombophlebitis, failure to wean off ventilator for >48 h, progressive renal insufficiency, acute renal failure, urinary tract infection, stroke or cerebral vascular accident, coma for >24 h, peripheral nerve injury, cardiac arrest, seizure, bleeding requiring transfusion, graft failure, or sepsis. Readmission data were also collected. Readmission was defined as admission after initial discharge within the first 30 postoperative days.

Information on medical comorbidities and other potential risk factors was obtained for each patient. Medical comorbidities included pulmonary, cardiac, nutritional, gastrointestinal (GI), central nervous system (CNS), history of prematurity, bleeding disorders, congenital malformation, seizures, and cerebral palsy. Pulmonary comorbidities included ventilator dependence, current pneumonia, asthma, cystic fibrosis, bronchopulmonary dysplasia, oxygen support, and structural pulmonary abnormality. Nutritional comorbidities included need for nutritional support and failure to thrive. GI comorbidities included esophageal, gastric, or intestinal disease and biliary, liver, or pancreatic disease. CNS comorbidities included history of stroke, coma, developmental delay, and acquired CNS abnormality. Other variables studied include race, gender, open fracture, weight-for-age, prolonged operative time, preoperative systemic inflammatory response syndrome, and an American Society of Anesthesiologist (ASA) score ≥3. In addition, we noted whether the procedure was performed by a surgeon under the specialty of “Pediatric Orthopedic Surgery” versus “Orthopedics” in order to discern any possible disparities attributable to differences in training.

Statistical analysis was performed using the SPSS software program (IBM Corp., Armonk, NY). Univariate analysis was performed using Fisher’s exact test to determine associations between preoperative and operative variables with AAE and readmission. Variables with a significance of *p* < 0.20 in the univariate analysis were then entered into a multivariate analysis using backwards stepwise binary logistic regression. In logistic regression, variables with a significance of *p* < 0.05 were determined to be independent risk factors.

## Results

A total of 522 patients who underwent operative fixation of a femur fracture with an intramedullary implant were included in the study. The mean age of patients was 10.2 ± 3.8 (range 4–18) years, 123 (23.6 %) of the patients were female, and 167 (32 %) were of a racial minority. Weight was available for 331 patients, of whom 84 (25.8 %) were obese. Obesity was defined as a weight-for-age of >95th percentile. Of the procedures, 447 (85.6 %) were performed by pediatric orthopedic surgeons.

Overall, 18 (3.4 %) patients sustained an AAE, among which the most common was bleeding requiring a transfusion. Table 1 summarizes the adverse events. Among the nine patients who required a transfusion, three had comorbidities which included neuromuscular disease, obesity, and asthma. The obese patient did undergo angiography with repair of a blood vessel. A fourth patient who required a transfusion had an open femur fracture.

**Table 1:** Short-term adverse events after operative fixation

Adverse event	Number of patients
Infection	4
Neurologic injury	1
Bleeding requiring transfusion	9
Deep venous thromboembolism	2
Pulmonary embolism	1
Wound dehiscence	1

Twenty patients (3.8 %) were readmitted, among whom 13 (2.5 %) required a reoperation. The most common reoperation procedure was removal of the implant. The reasons for readmission after the operative fixation procedure and the reoperation procedures are summarized in Tables 2 and 3, respectively.

**Table 2:** Reasons for readmission after operative fixation

Reason for readmission	Number of patients
Deep venous thromboembolism	2
Wound disruption	2
Acute postoperative pain	1
Malalignment of fracture	1
Fracture of femur	2
Mechanical complication of orthopedic device	1
Other complications due to orthopedic device	1
Postoperative infection	1
Reason not documented	9

**Table 3:** Reasons for reoperation after operative fixation

Reoperation procedure	Number of patients
Removal of implant, deep	5
Open treatment of femoral shaft fracture, with or without external fixation, with insertion of intramedullary implant, with or without cerclage and/or locking screws	3
Repair, nonunion or malunion, femur, distal to head and neck; without graft (e.g., compression technique)	1
Closed treatment of femoral shaft fracture, with manipulation, with or without skin or skeletal traction	1
Open treatment of femoral shaft fracture with plate/screws, with or without cerclage	1
Thoracoscopy, diagnostic (separate procedure); mediastinal space, with biopsy	1
Embolectomy or thrombectomy, with or without catheter; femoropopliteal, aortoiliac artery, by leg incision	1

We first performed a univariate analysis, which revealed that the factors associated with AAE included a cardiac comorbidity (*p* = 0.021), prolonged operative time (*p* < 0.001), open fracture (*p* = 0.035), and female gender (*p* = 0.046). Prolonged operative time was defined as one standard deviation (SD) above the mean operative time. The mean operative time for patients in the study was 93.8 (SD 53.2) min. Factors associated with readmission included a GI comorbidity (*p* = 0.043), CNS comorbidity (*p* = 0.001), nutritional comorbidity (*p* = 0.007), seizure disorder (*p* = 0.002), and cerebral palsy (*p* = 0.007).

We then performed a multivariate analysis, which revealed that independent risk factors for AAE were a cardiac comorbidity [*p* = 0.018, odds ratio (OR) 12.7, 95 % confidence interval (CI) 1.5, 103.7], open fracture (*p* = 0.022, OR 10.2, 95 % CI 1.4, 74.4), and prolonged operative time (*p* < 0.001, OR 17.5, 95 % CI 6.1, 50.5). Independent risk factors for readmission were a CNS comorbidity (*p* = 0.021, OR 4.5, 95 % CI 1.3, 16.2) and seizure disorder (*p* = 0.048, OR 4.9, 95 % CI 1.0, 23.5). Table 4 summarizes the independent risk factors for AAE and readmission.

**Table 4:** Independent risk factors for any adverse events and readmission

Risk factor	Significance (*p*)	Odds ratio (95 % CI)
Any adverse event (AAE)		
Cardiac comorbidity	0.018	12.7 (1.5, 103.7)
Open fracture	0.022	10.2 (1.4, 74.4)
Prolonged operative time (>147 min)	0.001	17.5 (6.1, 50.5)
Readmission		
CNS comorbidity	0.021	4.5 (1.3, 16.2)
Seizure disorder	0.048	4.9 (1.0, 23.5)

CI, Confidence interval; CNS, central nervous system

## Discussion

Operative fixation of pediatric femur fractures with intramedullary implants continues to be a commonly performed procedure and overall is considered to be safe. Previous studies have documented complications and their risk factors but have largely focused on hardware prominence and fracture union and alignment characteristics [1, 3, 4, 15–17]. No previous studies have identified risk factors for short-term adverse events and readmission. The purpose of this study was to use a large, nationally representative database to determine which factors contribute to poor outcomes in the 30-day postoperative period.

The data which we analyzed suggest that a cardiac comorbidity, an open fracture, and a prolonged operative time are independent risk factors for AAE. Specifically, open fractures have previously been shown to increase adverse events [18]. Our analyses also identified a CNS comorbidity or a seizure disorder as independent risk factors for readmission. Both CNS abnormalities and seizure disorders have been shown to increase short-term morbidity and increase readmission rates in pediatric spinal surgery [19, 20]. It should be noted that for two patients included in our study, the reason for readmission was coded as “femur fracture”. We feel that this likely represents an error in coding and that the true reason for readmission was due to another issue related to the patient’s original diagnosis of femur fracture.

The most common AAE among our patient cohort was bleeding requiring a transfusion. Although blood transfusions are generally considered to be safe, numerous concerns still remain. Blood transfusions pose unique risks to the pediatric population. Specifically, the incidence of acute transfusion reactions, usually allergic in nature, is much higher for children than adults. Also, in sickle cell or oncology pediatric patients, iron overload syndrome is worrisome [21]. Thus, blood transfusions in pediatric patients must be utilized cautiously with close monitoring.

Neither obesity nor age were significant risk factors for AAE or readmission. Both obesity and age have been shown to be predictors for poor outcomes after operative fixation of pediatric femurs in previous studies [3, 4]. In addition, obesity and age have been linked to an increased risk for the development of venous thromboembolism in pediatric patients undergoing orthopedic surgery [22]. However, in our study these variables did not increase the incidence of AAE or readmission. A limiting factor in the study was that weight data were available for only 331 patients. The rate of obesity in our study was 25.8 %, which is higher than the 16.9 % rate found in the general population of children and adolescents in the USA [23]. A previous study on pediatric patients sustaining lower extremity fractures documented an obesity rate of 18.4 % [24]. Thus, our obesity rate seems to be slightly higher than that reported by other studies. Longer term studies and better data collection are needed to further evaluate the effect of these variables on clinical outcomes.

With recent healthcare reforms, a greater emphasis has been placed on physicians’ effectiveness and whether specialists provide a better quality of care and improved outcomes. A previous study demonstrated that specialized surgeons produce better outcomes than general surgeons after general thoracic surgical procedures [25]. In another study, adverse events after complicated appendicitis were significantly more prevalent for those who were treated by general surgeons than by pediatric surgeons [26]. In our study, however, no differences in short-term adverse events or readmission were found between pediatric and nonpediatric orthopedic surgeons. Similarly, another study showed that outcomes in children with supracondylar humerus fractures were comparable when treated by either a pediatric or nonpediatric orthopedic surgeon [27]. However, it should be noted that, according to the ACS, procedures were coded under “Pediatric Orthopedic” or “Orthopedic” by a surgical clinical reviewer. Thus, a potential for error is present when choosing a coding label for the surgeon performing the procedure.

The weaknesses of the study are related to the limitations of the ACS NSQIP database. First, multiple variables are not collected in the database, such as insurance status, hospital volume, surgeon volume, Glasgow Coma Scores, Injury Severity Scores (ISSs), and complications or readmission after the first 30 postoperative days. High ISSs would have been an important variable to study as a risk factor for complications but were not available. However, we did perform a subanalysis with regard to other injuries treated surgically in patients undergoing operative fixation of a femur fracture and found that having a concurrent surgery was not a risk factor for AAE or readmission. Second, we were unable to study radiographic outcomes in relation to malunion or hardware prominence. Third, the CPT code for the operative fixation of a pediatric femur fracture includes both retrograde elastic nails and rigid antegrade nails. One previous study has shown that outcomes with pediatric femur fractures are improved when elastic nailing is limited to stable femur fractures and trochanteric entry nails are utilized for unstable femur fractures [5]. There may have been variability in the material of the elastic nail. A recent study, however, showed no significant differences between titanium and stainless steel elastic nails with regard to complications, fracture healing, and radiologic angulation [28].

Despite these weaknesses in our study, we have identified risk factors for short-term adverse events and readmission. Surgeons can utilize this information during preoperative counseling. Furthermore, hospitals may be able to target patients who are at increased risk for short-term adverse events or readmission and implement measures which would help address and decrease such occurrences.

## References

[1] Flynn J, Hresko T, Reynolds R (2001). Titanium elastic nails for pediatric femur fractures: a multicenter study of early results with analysis of complications. J Pediatr Orthop.

[2] Hosalkar H, Pandya N, Cho R (2011). Intramedullary nailing of pediatric femoral shaft fracture. J Am Acad Orthop Surg.

[3] Luhmann S, Schootman M, Schoenecker P (2003). Complications of titanium elastic nails for pediatric femoral shaft fractures. J Pediatr Orthop.

[4] Moroz L, Launay F, Kocher M (2006). Titanium elastic nailing of fractures of the femur in children. Predictors of complications and poor outcome. J Bone Joint Surg Br.

[5] Sink E, Faro F, Polousky J (2010). Decreased complications of pediatric femur fractures with a change in management. J Pediatr Orthop.

[6] Heffernan M, Gordon J, Sabatini C (2014). Treatment of femur fractures in young children: a multicenter comparison of flexible intramedullary nails to spica casting in young children aged 2 to 6 years. J Pediatr Orthop.

[7] Karaman I, Halici M, Kafadar I (2014). Mid-term results of the elastic intramedullary nailing in paediatric long bone shaft fractures: a prospective study of 102 cases. J Pediatr Orthop B.

[8] Baldwin K, Hsu J, Wenger D (2011). Treatment of femur fractures in school-aged children using elastic stable intramedullary nailing: a systematic review. J Pediatr Orthop B.

[9] Reynolds R, Legakis J, Thomas R (2012). Intramedullary nails for pediatric diaphyseal femur fractures in older, heavier children: early results. J Child Orthop.

[10] Jalan D, Chandra R, Sharma V (2013). Results of titanium elastic nailing in paediatric femoral diaphyseal fractures—report of 30 cases. Chin J Traumatol..

[11] Sun L, Yang J, Tian N (2014). Pediatric femoral shaft fractures treated with titanium elastic nailing. Orthopedics..

[12] Kirby R, Winquist R, Hansen S (1981). Femoral shaft fractures in adolescents: a comparison between traction plus cast treatment and closed intramedullary nailing. J Pediatr Orthop.

[13] Reeves R, Ballard R, Hughes J (1990). Internal fixation versus traction and casting of adolescent femoral shaft fractures. J Pediatr Orthop.

[14] Parikh S, Jain V, Denning J (2012). Complications of elastic stable intramedullary nailing in pediatric fracture management. Bone Joint Surg Am.

[15] Narayanan U, Hyman J, Wainwright A (2004). Complications of elastic stable intramedullary nail fixation of pediatric femoral fractures, and how to avoid them. J Pediatr Orthop.

[16] Salonen A, Lahdes-Vasama T, Mattila V (2014). Pitfalls of femoral titanium elastic nailing. Scand J Surg.

[17] Lascombes P, Nespola A, Poircuitte J (2012). Early complications of with flexible intramedullary nailing in childhood fracture: a 100 cases managed with precurved tip and shaft nails. Orthop Traumatol Surg Res.

[18] Flynn J, Schwend R (2004). Management of pediatric femoral shaft fractures. J Am Acad Orthop Surg.

[19] Pugely A, Martin C, Gao Y (2014). The incidence and risk factors for short-term morbidity and mortality in pediatric deformity spinal surgery: an analysis of the NSQIP pediatric database. Spine.

[20] Martin CT, Pugely AJ, Gao Y, Weinstein SL (2015). Causes and risk factors for 30 day unplanned readmissions after pediatric spinal deformity surgery. Spine.

[21] Lavoie J (2011). Blood transfusion risks and alternative strategies in pediatric patients. Paediatr Anaesth.

[22] Georgopoulos G, Hotchkiss M, McNair B et al (2015) Incidence of deep vein thrombosis and pulmonary embolism in the elective pediatric orthopaedic patient. J Pediatr Orthop. doi: 10.1097/BPO.0000000000000391 10.1097/BPO.0000000000000391 PMC4496329 25575361

[23] Ogden CL, Carroll MD, Kit BK, Flegal KM (2012). Prevalence of obesity and trends in body mass index among US children and adolescents, 1999-2010. JAMA.

[24] Gilbert SR, MacLennan PA, Backstrom I, Creek A, Sawyer J (2015). Altered lower extremity fracture characteristics in obese pediatric trauma patients. J Orthop Trauma.

[25] Schipper P, Diggs B, Ungerleider R (2009). The influence of surgeon specialty on outcomes in general thoracic surgery: a national sample 1996 to 2005. Ann Thorac Surg.

[26] da Silva P, de Aguiar V, Waisberg J (2014). Pediatric surgeon vs general surgeon: does subspecialty training affect the outcome of appendicitis?. Pediatr Int.

[27] Farley F, Patel P, Craig C (2008). Pediatric supracondylar humerus fractures: treatment by type of orthopedic surgeon. J Child Orthop.

[28] Goyal N, Aggarwal A, Mishra P (2014). Randomized controlled trial comparing stabilization of fresh close femoral shaft fractures in children with titanium elastic nail system versus stainless steel elastic nail system. Acta Orthop Belg.

